# Medical dispensation in La Rioja, Spain: epidemiology and its relationship with social determinants: a descriptive study

**DOI:** 10.3389/fphar.2025.1667148

**Published:** 2025-11-03

**Authors:** Gonzalo Aparicio-Rodríguez, Raúl Juárez-Vela, Amaya Burgos-Esteban, Consuelo Sancho-Sánchez, José Ángel Santos-Sanchez, Noelia Navas-Echazarreta, Ana Cobos-Rincón, Antonio Cardoso-Muñoz, Regina Ruiz de Viñaspre-Hernández, Ignacio M. Larrayoz

**Affiliations:** ^1^ Interuniversity Doctoral Program in Global Health by the University of Zaragoza, the University of La Rioja, the Public University of Navarre, and the University of Lleida, Logroño, La Rioja, Spain; ^2^ GRUPAC, Research Group in Care, University of La Rioja, La Rioja, Spain; ^3^ Department of Nursing, Faculty of Health Sciences, University of La Rioja, La Rioja, Spain; ^4^ Intensive Care Unit, University Hospital San Pedro, Logroño, La Rioja, Spain; ^5^ Department of Physiology and Pharmacology, Faculty of Medicine, University of Salamanca, Salamanca, Spain; ^6^ Department of Biomedical Sciences and Diagnosis, Faculty of Medicine, University of Salamanca, Salamanca, Spain; ^7^ Department of Nursing and Physiotherapy, Faculty of Nursing, University of Salamanca, Salamanca, Spain

**Keywords:** chronic disease, drug prescriptions, anatomical therapeutic chemical classificationsystem, social determinants, socioeconomic factors

## Abstract

**Introduction:**

Spain stands out as the European Union country with the highest life expectancy, reaching 83.2 years in 2022. This context is accompanied by population aging and an increase in chronic and degenerative diseases, which translates into greater medication use. In 2022, the National Health System (NHS) dispensed over 1,127.8 million packages. This study aims to evaluate the state of medication dispensation in La Rioja and its relationship with health determinants such as economic conditions, area of residence, age, and gender.

**Methods:**

We conducted an observational, retrospective, cross-sectional study between January 2016 and December 2023. A total of 4,108,656 raw e-dispensations (2016–2023) were recorded, from which 1,433,531 unique patient–ATC4–year records (26 frequent subgroups) were analyzed. We included patients aged 14 years and older with electronic dispensations. Variables analyzed included age, gender, socioeconomic level, type and number of dispensations, and the patient’s basic health zone. Statistical analyses employed Chi-square tests for categorical associations and Kruskal–Wallis tests to compare age distributions across ATC4 medication groups, with a significance level of p < 0.05.

**Results:**

Proton pump inhibitors (PPIs) were the most dispensed medications in La Rioja, with 82,195 dispensations between 2016 and 2023, followed by propionic acid derivative anti-inflammatory drugs. Antidepressant dispensations increased from 5,281 in 2016 to 7,486 in 2023. Regarding gender differences, women accounted for more dispensations (53.7%). The largest differences favoring women were observed in thyroid hormones, vitamin D, and antidepressant groups. Conversely, medication groups indicated for cardiovascular pathology—such as platelet aggregation inhibitors and angiotensin-converting enzyme inhibitors—showed a significant difference favoring men. Among the elderly, the most dispensed medications also corresponded to families indicated for cardiovascular diseases. By health zones, PPI dispensation was high and homogeneous in the Rioja 1 and Rioja 2 clusters, while anxiolytics and antidepressants stood out in the municipality of San Román and the Guindalera area of Logroño. In socioeconomic terms, pensioners with limited incomes (IHC 002) primarily consumed PPIs, paracetamol, and benzodiazepines, while low-income workers (IHC 003) showed notable dispensation of propionic acid derivatives, PPIs, and paracetamol.

**Discussion:**

Our findings align with national and European trends: PPIs and propionic acid derivative anti-inflammatories are the most frequently dispensed medications. Between 2016 and 2023, we observed an increase in the absolute number of unique users in ATC4 subgroup N06AX (Other antidepressants) (+41.8%) and, to a lesser extent, in N06AB (Selective serotonin reuptake inhibitors) (+25.5%), while the annual relative share of N06AB remained essentially stable. Socioeconomic determinants—such as low income and unemployment—appear to directly influence access to and dispensation of medications.

**Conclusion:**

Medication dispensation patterns in La Rioja mirror broader national and EU trends, with PPIs and propionic acid derivatives leading. Gender, age, geographic zone, and socioeconomic status are associated with distinct dispensation profiles. Targeted public health strategies should consider these determinants to optimize rational medication use and equity in access.

## 1 Introduction

Spain positions itself as the European Union country with the highest life expectancy, reaching 83.2 years in 2022 (INE, 2025). Additionally, a favorable trend has been observed following the significant decline between 2019 and 2020 due to the COVID-19 pandemic (OECD, 2023). The leading causes of mortality are cardiovascular diseases and cancer, which together account for more than 50% of deaths (Ministerio de Sanidad, 2023a). In the coming years, the national landscape is expected to be shaped by an increasingly aging population, a higher prevalence of chronic and degenerative diseases, and, consequently, greater use of medications (La Consejería de Salud y, 2017).

Medications play a fundamental role in healthcare, serving as essential tools for health professionals in modifying the natural course of a disease, its prevention, or diagnosis. Medication dispensation is the most frequent medical intervention within the doctor–patient relationship (CAP1311, 2025). Ensuring the rational use of medications is therefore crucial to improving health outcomes, increasing quality of life, and sustaining pharmaceutical services (La Consejería de Salud y, 2017). The World Health Organization (WHO) expert conference held in Nairobi in 1985 defined the concept of “rational use of medicines” as the situation in which patients receive medications appropriate to their clinical needs, in doses that correspond to their individual requirements, for an adequate period, and at the lowest cost possible to them and the community (World Health Organization WHO, 2025). Older adults represent the population group with the highest medication dispensation, primarily due to the increase in health problems associated with aging (Martin-Pérez et al., 2016).

At the national level, 1,127.8 million packages of medications were dispensed through National Health System (NHS) dispensations at pharmacies in 2022, generating public pharmaceutical spending of €12.801 billion, a 4.9% increase compared to the previous year (Ministerio de Sanidad, 2023a). In La Rioja, during 2022, a total of 6,970,466 packages of medications were dispensed through NHS dispensations at community pharmacies, resulting in pharmaceutical spending of €82,057,393, a 5.6% increase compared to 2021 (Consejería de Salud y Políticas Sociales del Gobierno de La Rioja, 2022).

Medications also serve as tools for evaluating medical practice; the choice of prescribed medications reflects medical knowledge, skills, and consideration of pharmaceutical efficiency by health professionals (CAP1311, 2025). The analysis of pharmaceutical treatments prescribed within an autonomous community, using dispensation data from the Regional Health Service, allows for the identification and correlation of potential inequalities based on territory, gender, and socioeconomic level (AtlasVPM, 2025). This approach facilitates the identification of the social determinants of health, which, according to the WHO, are the circumstances in which people are born, grow, work, live, and age, as well as the broader set of forces and systems that influence daily living conditions (PAHO, 2023). The amount of data showing biological and gender differences in the field of medicine is increasing significantly; however, it is still necessary to investigate how medications are used differently between genders across all age groups (Roe et al., 2002).

Despite the relevance of these determinants, there is no updated, equity-focused mapping of differential use of ATC4 therapeutic subgroups in La Rioja that integrates demographic, territorial, and socioeconomic dimensions. Leveraging the regional dispensation registry (AtlasVPM, 2025), this study addresses that gap. The analytical unit is defined as one record per patient–ATC4 subgroup–year for users aged ≥14 years, restricting to a single annual event per individual and subgroup to avoid duplication.

The objective of this research is to evaluate the state of medication dispensation in La Rioja and establish its relationship with health determinants, such as economic conditions, area of residence, age, and gender.

## 2 Materials and methods

### 2.1 Study design

An observational, retrospective, and cross-sectional study was conducted between January 2016 and December 2023.

### 2.2 Population and scope of the study

The study was conducted in the Autonomous Community of La Rioja (Spain). The analytical sample consisted of 4,108,656 records retrieved from the Data Unit of the Government of La Rioja, representing the complete set of dispensations, encompassing both public sectors made by pharmacies in the region during the period from 2016 to 2023. After a data cleansing process aimed at identifying the most frequently dispensed medications, each final record represented a patient who had been dispensed a medication belonging to a subgroup of level 4 of the Anatomical, Therapeutic, Chemical classification system (ATC4). The 26 most frequent ATC4 subgroups were identified, resulting in a total of 1,433,531 records. This analysis of the 26 most frequent ATC4 subgroups was conducted based on the prior assessment used to identify the most frequently dispensed ATC4 groups.

The inclusion criteria encompassed all pharmacy dispensations in La Rioja recorded through the electronic dispensing system, considering only patients aged 14 years or older during the study period. In the Spanish National Health System, individuals under 14 years of age are classified as a pediatric population.

A single dispensation per ATC group/patient/year was considered. The exclusion criteria were incomplete data and dispensations corresponding to individuals under 14 years of age. This study was approved by the Ethics Committee of La Rioja (CeimLar) with protocol N° 750.

### 2.3 Variables

Sociodemographic data were obtained from electronic records. These characteristics included age, gender, socioeconomic level, type of dispensation, and the basic health zone.

### 2.4 Statistical analysis

Quantitative variables were summarized using the mean and standard deviation (SD) when the data followed a normal distribution, and the median and interquartile range (IQR) when they did not. The mean and SD were specifically calculated to describe the average age in each ATC medication group. Categorical variables were analyzed using absolute frequencies and percentages, which allowed for the description of the distribution of medications (ATC) by year, sex, and basic health zone.

For statistical analysis, the Chi-square test was used to evaluate significant relationships between categorical variables, such as the association between ATC codes and year, ATC codes and sex, and ATC codes and basic health zone. This test was deemed appropriate due to the sufficient sample size. To compare the distributions of the variable “age” among the different ATC medication groups, the Kruskal-Wallis test was applied.

Additionally, cross-tabulations were generated to explore relationships between categorical variables, such as ATC codes and sex, and ATC codes and geographic area, reporting absolute counts and column percentages All statistical analyses were performed using STATA/SE v.21.0 software (College Station, Texas, United States), establishing a significance level of p < 0.05. Sociodemographic data were obtained from electronic records. These characteristics included age, gender, employment status, socioeconomic level, type and number of dispensations, and basic health zone.

## 3 Results

Between 2016 and 2023, the total annual number of dispensations for the main ATC medication groups included in the table shows a general upward trend. In 2016, 160,291 dispensations were recorded, progressively increasing to 207,053 in 2023. This increase is consistent throughout the analyzed years, except for 2020, the year the COVID-19 pandemic began. Regarding the distribution by ATC groups, some groups exhibit consistently high dispensation charts across all years. For instance, the A02BC group (Proton pump inhibitors) maintains values above 9,400 annual dispensations, reaching 11,443 in 2023. Similarly, the M01AE group (Propionic acid derivatives), N02BE group (Anilides), and N05BA group (Benzodiazepine derivatives. Anxiolytics) also show high absolute numbers.

**FIGURE 1:**
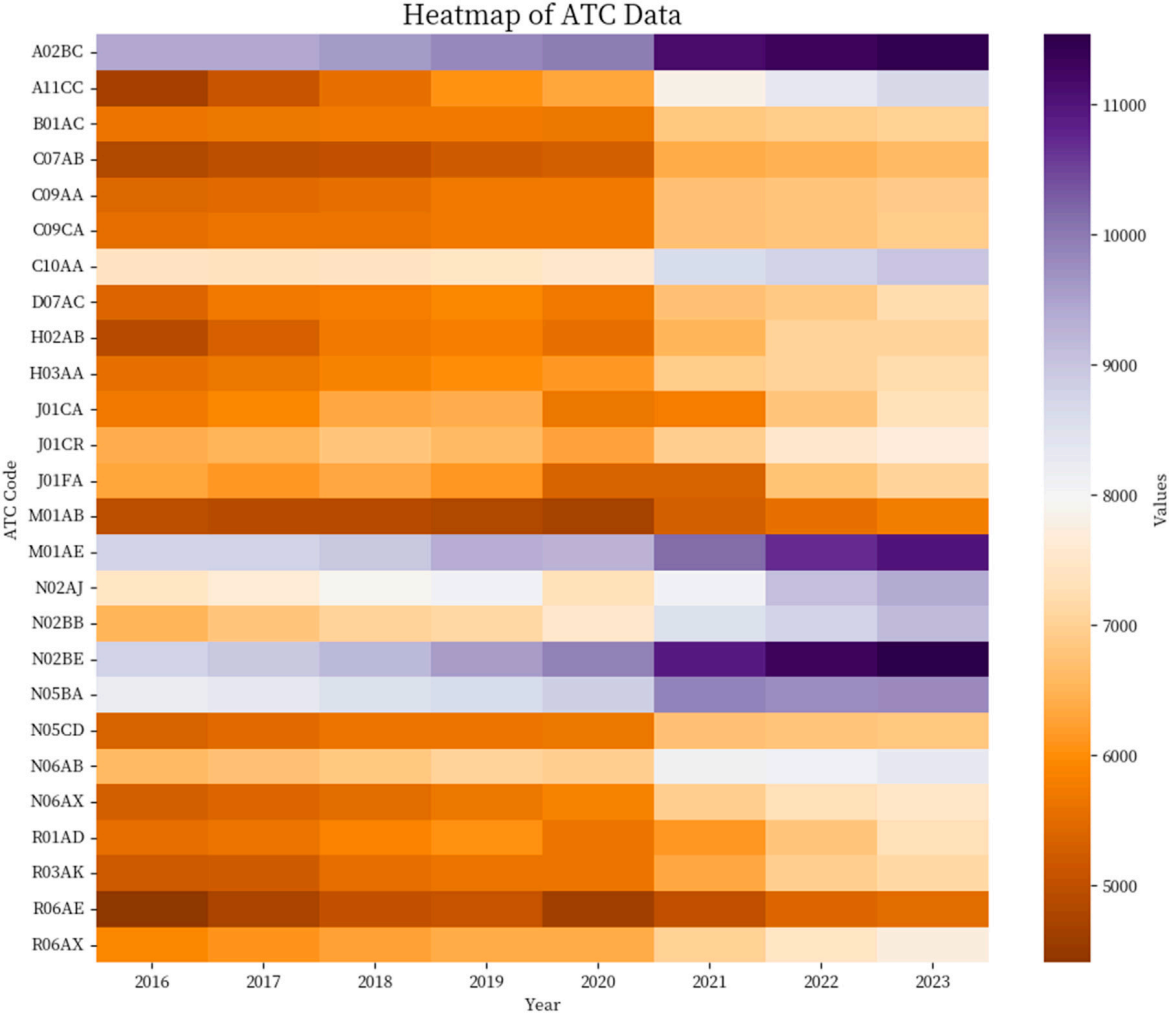
Dispensation evolution 2016–2023.

In terms of the increase in the number of dispensations from 2016 to 2023, a notable rise is observed in Vitamin D and analogues (A11CC), followed by Glucocorticoids (H02AB) and Other Antidepressants (N06AX). On the other hand, the macrolide antibiotics family shows a very contained increase. See Table 1 and Figure 1.

**TABLE 1:** Dispensation evolution 2016–2023.

ATC	2016	2017	2018	2019	2020	2021	2022	2023	Total
A02BC Proton pump inhibitors	9,443 (5.89%)	9,433 (5.76%)	9,608 (5.70%)	9,812 (5.72%)	9,973 (5.89%)	11,152 (5.83%)	11,331 (5.62%)	11,443 (5.53%)	82,195 (5.73%)
A11CC Vitamin D and analogues	4,682 (2.92%)	5,119 (3.13%)	5,595 (3.32%)	6,075 (3.54%)	6,305 (3.72%)	7,818 (4.08%)	8,344 (4.14%)	8,647 (4.18%)	52,585 (3.67%)
B01AC Platelet aggregation inhibitors, excl. Heparin	5,660 (3.53%)	5,666 (3.46%)	5,714 (3.39%)	5,730 (3.34%)	5,678 (3.35%)	6,846 (3.58%)	6,922 (3.43%)	7,013 (3.39%)	49,229 (3.43%)
C07AB Beta blocking agents, selective	4,818 (3.01%)	4,965 (3.03%)	5,046 (2.99%)	5,219 (3.04%)	5,289 (3.12%)	6,397 (3.34%)	6,478 (3.21%)	6,623 (3.20%)	44,835 (3.13%)
C09AA ACE inhibitors, plain	5,429 (3.39%)	5,493 (3.36%)	5,563 (3.30%)	5,715 (3.33%)	5,740 (3.39%)	6,729 (3.51%)	6,822 (3.38%)	6,912 (3.34%)	48,403 (3.38%)
C09CA Angiotensin II receptor blockers	5,546 (3.46%)	5,609 (3.43%)	5,616 (3.33%)	5,724 (3.34%)	5,734 (3.39%)	6,719 (3.51%)	6,800 (3.37%)	6,929 (3.35%)	48,677 (3.40%)
C10AA HMG CoA reductase inhibitors	7,414 (4.63%)	7,363 (4.50%)	7,409 (4.39%)	7,469 (4.35%)	7,531 (4.45%)	8,596 (4.49%)	8,763 (4.35%)	8,993 (4.34%)	63,538 (4.43%)
D07AC Corticosteroids, potent (group III)	5,395 (3.37%)	5,733 (3.50%)	5,754 (3.41%)	5,917 (3.45%)	5,712 (3.37%)	6,719 (3.51%)	6,864 (3.40%)	7,230 (3.49%)	49,324 (3.44%)
H02AB Glucocorticoids	4,890 (3.05%)	5,300 (3.24%)	5,730 (3.40%)	5,819 (3.39%)	5,580 (3.30%)	6,531 (3.41%)	7,033 (3.49%)	7,059 (3.41%)	47,942 (3.34%)
H03AA Thyroid hormones	5,561 (3.47%)	5,681 (3.47%)	5,869 (3.48%)	6,001 (3.50%)	6,147 (3.63%)	6,916 (3.61%)	7,037 (3.49%)	7,200 (3.48%)	50,412 (3.52%)
J01CA Penicillin with extended spectrum	5,703 (3.56%)	5,928 (3.62%)	6,339 (3.76%)	6,415 (3.74%)	5,679 (3.35%)	5,752 (3.00%)	6,808 (3.38%)	7,312 (3.53%)	49,936 (3.48%)
J01CR Combinations of penicillins, incl. Beta lactamase inhibitors	6,436 (4.02%)	6,514 (3.98%)	6,821 (4.05%)	6,614 (3.86%)	6,281 (3.71%)	6,945 (3.63%)	7,544 (3.74%)	7,699 (3.72%)	54,854 (3.83%)
J01FA Macrolides	6,320 (3.94%)	6,146 (3.76%)	6,341 (3.76%)	6,108 (3.56%)	5,364 (3.17%)	5,335 (2.79%)	6,771 (3.36%)	7,064 (3.41%)	49,449 (3.45%)
M01AB acetic acid derivatives and related substances	4,952 (3.09%)	4,927 (3.01%)	4,891 (2.90%)	4,807 (2.80%)	4,709 (2.78%)	5,297 (2.77%)	5,584 (2.77%)	5,784 (2.79%)	40,951 (2.86%)
M01AE Propionic acid derivatives	8,764 (5.47%)	8,790 (5.37%)	8,964 (5.32%)	9,347 (5.45%)	9,262 (5.47%)	10,163 (5.31%)	10,718 (5.32%)	10,998 (5.31%)	77,006 (5.37%)
N02AJ Opioids in combination with non-opioid analgesics	7,454 (4.65%)	7,612 (4.65%)	7,907 (4.69%)	8,039 (4.69%)	7,304 (4.31%)	8,036 (4.20%)	9,099 (4.51%)	9,404 (4.54%)	64,855 (4.52%)
N02BB Pyrazolones	6,550 (4.09%)	6,822 (4.17%)	7,047 (4.18%)	7,109 (4.14%)	7,499 (4.43%)	8,499 (4.44%)	8,769 (4.35%)	9,143 (4.42%)	61,438 (4.29%)
N02BE Anilides	8,795 (5.49%)	8,953 (5.47%)	9,191 (5.45%)	9,569 (5.58%)	9,893 (5.84%)	10,913 (5.70%)	11,285 (5.60%)	11,532 (5.57%)	80,131 (5.59%)
N05BA Benzodiazepine derivatives. Anxiolytics	8,227 (5.13%)	8,315 (5.08%)	8,514 (5.05%)	8,629 (5.03%)	8,818 (5.21%)	9,864 (5.15%)	9,769 (4.85%)	9,796 (4.73%)	71,932 (5.02%)
N05CD Benzodiazepine derivatives. Hypnotics and sedatives	5,334 (3.33%)	5,480 (3.35%)	5,653 (3.35%)	5,636 (3.29%)	5,684 (3.36%)	6,722 (3.51%)	6,792 (3.37%)	6,855 (3.31%)	48,156 (3.36%)
N06AB Selective serotonin reuptake inhibitors	6,590 (4.11%)	6,738 (4.12%)	6,854 (4.07%)	7,048 (4.11%)	6,979 (4.12%)	8,090 (4.23%)	8,123 (4.03%)	8,268 (3.99%)	58,690 (4.09%)
N06AX Other antidepressants	5,281 (3.29%)	5,391 (3.29%)	5,498 (3.26%)	5,670 (3.30%)	5,862 (3.46%)	6,957 (3.63%)	7,298 (3.62%)	7,486 (3.62%)	49,443 (3.45%)
R01AD Corticosteroids	5,537 (3.45%)	5,620 (3.43%)	5,828 (3.46%)	6,024 (3.51%)	5,656 (3.34%)	6,119 (3.20%)	6,827 (3.39%)	7,278 (3.52%)	48,889 (3.41%)
R03AK Adrenergics in combination with corticosteroids or other drugs, excl. Anticholinergics	5,177 (3.23%)	5,229 (3.20%)	5,541 (3.29%)	5,619 (3.28%)	5,636 (3.33%)	6,330 (3.31%)	6,961 (3.45%)	7,157 (3.46%)	47,650 (3.32%)
R06AE Piperazine derivatives	4,408 (2.75%)	4,727 (2.89%)	5,047 (2.99%)	5,050 (2.94%)	4,613 (2.72%)	4,994 (2.61%)	5,397 (2.68%)	5,503 (2.66%)	39,739 (2.77%)
R06AX Other antihistamines for systemic use	5,925 (3.70%)	6,104 (3.73%)	6,257 (3.71%)	6,395 (3.73%)	6,388 (3.77%)	7,011 (3.66%)	7,467 (3.70%)	7,725 (3.73%)	53,272 (3.72%)
Total	160,291 (100%)	163,658 (100%)	168,597 (100%)	171,560 (100%)	169,316 (100%)	191,450 (100%)	201,606 (100%)	207,053 (100%)	1,433,531 (100%)

In the analysis of the results in terms of age, the mean values representing the average age of patients consuming specific medications and the standard deviation indicating the variability of ages within each group are presented. The medications with the highest average ages are those belonging to the subgroup B01AC (Platelet aggregation inhibitors, excl. Heparin) 66.41 ± 17.06, followed by medications in the subgroup C07AB (Beta blocking agents, selective) 65.93 ± 16.16 and the subgroup C09CA (Angiotensin II receptor blockers) 65.62 ± 15.88.

On the contrary, the medications with the lowest average ages are those belonging to the subgroup M01AE (Propionic acid derivatives) 50.40 ± 20.57, R06AX (Other antihistamines for systemic use) 50.78 ± 21.0650.78 ± 21.06, and J01CA (Penicillin with extended spectrum) 51.65 ± 21.26.

It is worth noting that the highest standard deviation is observed in the subgroup N02BE (Anilides) 22.1322.13, indicating greater dispersion in the ages of the patients using these medications.

The Rank Sum, which allows for evaluating the age distribution within each group, shows that medications such as proton pump inhibitors and analgesics like paracetamol, which have a high number of observations, also present high Rank Sum values, indicating they cover a wide range of ages.

To determine whether there are significant differences in average ages among the different medication groups, the Kruskal-Wallis test was applied. The results showed a Chi-squared value of 73,572.660 with 25 degrees of freedom and a p-value of 0.0001, indicating statistically significant differences in average ages among the different medication groups. See Table 2.

**TABLE 2:** Analysis of ATC and average ages.

ATC category	Mean	Standard deviation	Observations	Rank sum
A02BC Proton pump inhibitors	58.58035	20.44247	82,195	6.05e+10
A11CC Vitamin D and analogues	60.31068	20.03484	52,585	4.07e+10
B01AC Platelet aggregation inhibitors, excl. Heparin	66.41392	17.06375	49,229	4.41e+10
C07AB Beta blocking agents, selective	65.93400	16.15923	44,835	3.98e+10
C09AA ACE inhibitors, plain	64.43549	16.43515	48,403	4.14e+10
C09CA Angiotensin II receptor blockers	65.61660	15.88317	48,677	4.28e+10
C10AA HMG CoA reductase inhibitors	64.25460	16.08962	63,538	5.40e+10
D07AC Corticosteroids, potent (group III)	54.93985	21.40075	49,324	3.31e+10
H02AB Glucocorticoids	57.28770	20.29733	47,942	3.42e+10
H03AA Thyroid hormones	57.71632	19.70559	50,412	3.64e+10
J01CA Penicillin with extended spectrum	51.65518	21.26336	49,936	3.03e+10
J01CR Combinations of penicillins, incl. Beta lactamase inhibitors	52.82091	21.57255	54,854	3.44e+10
J01FA Macrolides	52.91699	21.12974	49,449	3.12e+10
M01AB acetic acid derivatives and related substances	54.10933	17.77480	40,951	2.64e+10
M01AE Propionic acid derivatives	50.40062	20.57034	77,006	4.47e+10
N02AJ Opioids in combination with non-opioid analgesics	55.56648	20.92701	64,855	4.42e+10
N02BB Pyrazolones	57.36752	20.26098	61,438	4.38e+10
N02BE Anilides	56.31673	22.13747	80,131	5.58e+10
N05BA Benzodiazepine derivatives. Anxiolytics	57.43416	20.00861	71,932	5.12e+10
N05CD Benzodiazepine derivatives. Hypnotics and sedatives	63.20947	18.19182	48,156	3.98e+10
N06AB Selective serotonin reuptake inhibitors	57.31014	20.20852	58,690	4.18e+10
N06AX Other antidepressants	60.85941	18.75304	49,443	3.85e+10
R01AD Corticosteroids	51.27074	20.39553	48,889	2.93e+10
R03AK Adrenergics in combination with corticosteroids or other drugs, excl. Anticholinergics	57.24451	20.77852	47,650	3.41e+10
R06AE Piperazine derivatives	51.25740	20.41135	39,739	2.37e+10
R06AX Other antihistamines for systemic use	50.77671	21.05693	53,272	3.14e+10

If we consider the distribution of medication dispensation classified by gender (men and women), the distribution is 46.3% for men (663,668) and 53.7% for women (769,863). Regarding gender differences, in several categories, dispensation was significantly higher in women than in men. For example, in H03AA (Thyroid hormones), 33,375 cases were recorded in women compared to 17,037 in men, representing almost double the proportion in women (4.34%) compared to men (2.57%). Another notable case is the A11CC group (Vitamin D and analogues), with 31,994 cases in women (4.16%) compared to 20,591 cases in men (3.10%), and N06AB (Selective Serotonin Reuptake Inhibitors), with 33,887 cases in women (4.40%) compared to 24,803 cases in men (3.74%).

**FIGURE 2:**
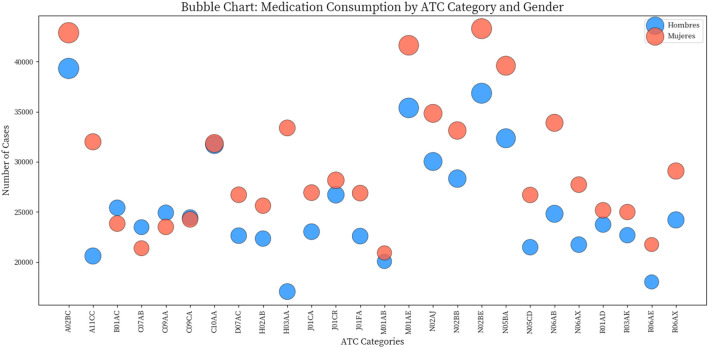
Bubble chart. Distribution of medication dispensation classified by ATC4 system and gender.

In contrast, some categories showed more balanced dispensation, such as opioid analgesics (N02AJ), where both men and women represented 4.52% of the total. It is worth highlighting medication groups used for cardiovascular pathologies, such as B01AC (Platelet aggregation inhibitors), C07AB (Selective beta blockers), and C09AA (ACE inhibitors), where the number of dispensations was higher in men. The Pearson chi2 value (7,700) with an associated probability of 0.000 confirms that there are statistically significant differences in the distribution of medication dispensation by gender and ATC category. See Table 3 and Figure 2.

**TABLE 3:** Distribution of medication dispensation classified by ATC4 system and gender.

ATC	H	M	Total
A02BC Proton pump inhibitors	39,316 (5.92%)	42,879 (5.57%)	82,195 (5.73%)
A11CC Vitamin D and analogues	20,591 (3.10%)	31,994 (4.16%)	52,585 (3.67%)
B01AC Platelet aggregation inhibitors, excl. Heparin	25,404 (3.83%)	23,825 (3.09%)	49,229 (3.43%)
C07AB Beta blocking agents, selective	23,469 (3.54%)	21,366 (2.78%)	44,835 (3.13%)
C09AA ACE inhibitors, plain	24,908 (3.75%)	23,495 (3.05%)	48,403 (3.38%)
C09CA Angiotensin II receptor blockers	24,434 (3.68%)	24,243 (3.15%)	48,677 (3.40%)
C10AA HMG CoA reductase inhibitors	31,707 (4.78%)	31,831 (4.13%)	63,538 (4.43%)
D07AC Corticosteroids, potent (group III)	22,621 (3.41%)	26,703 (3.47%)	49,324 (3.44%)
H02AB Glucocorticoids	22,329 (3.36%)	25,613 (3.33%)	47,942 (3.34%)
H03AA Thyroid hormones	17,037 (2.57%)	33,375 (4.34%)	50,412 (3.52%)
J01CA Penicillin with extended spectrum	23,019 (3.47%)	26,917 (3.50%)	49,936 (3.48%)
J01CR Combinations of penicillins, incl. Beta lactamase inhibitors	26,703 (4.02%)	28,151 (3.66%)	54,854 (3.83%)
J01FA Macrolides	22,577 (3.40%)	26,872 (3.49%)	49,449 (3.45%)
M01AB acetic acid derivatives and related substances	20,051 (3.02%)	20,900 (2.71%)	40,951 (2.86%)
M01AE Propionic acid derivatives	35,377 (5.33%)	41,629 (5.41%)	77,006 (5.37%)
N02AJ Opioids in combination with non-opioid analgesics	30,025 (4.52%)	34,830 (4.52%)	64,855 (4.52%)
N02BB Pyrazolones	28,316 (4.27%)	33,122 (4.30%)	61,438 (4.29%)
N02BE Anilides	36,842 (5.55%)	43,289 (5.62%)	80,131 (5.59%)
N05BA Benzodiazepine derivatives. Anxiolytics	32,344 (4.87%)	39,588 (5.14%)	71,932 (5.02%)
N05CD Benzodiazepine derivatives. Hypnotics and sedatives	21,470 (3.24%)	26,686 (3.47%)	48,156 (3.36%)
N06AB Selective serotonin reuptake inhibitors	24,803 (3.74%)	33,887 (4.40%)	58,690 (4.09%)
N06AX Other antidepressants	21,725 (3.27%)	27,718 (3.60%)	49,443 (3.45%)
R01AD Corticosteroids	23,731 (3.58%)	25,158 (3.27%)	48,889 (3.41%)
R03AK Adrenergics in combination with corticosteroids or other drugs, excl. Anticholinergics	22,670 (3.42%)	24,980 (3.24%)	47,650 (3.32%)
R06AE Piperazine derivatives	18,003 (2.71%)	21,736 (2.82%)	39,739 (2.77%)
R06AX Other antihistamines for systemic use	24,196 (3.65%)	29,076 (3.78%)	53,272 (3.72%)
Total	663,668 (100.00%)	769,863 (100.00%)	1,433,531 (100.00%)

The analysis of medication dispensation distribution in the cluster named RIOJA-1, composed of the basic health zones of Cervera, Alfaro, Calahorra, Arnedo, Murillo, San Román, Alberite, Torrecilla, and Navarrete, shows that Proton Pump Inhibitors (PPIs, A02BC) lead dispensation in most municipalities, with percentages ranging between 5% and 9% of total dispensations. The highest absolute values are recorded in Calahorra (5,509), followed by Arnedo (4,647) and Alberite (4,643). In municipalities with lower dispensation rates, such as San Román (692), the charts are smaller but still significant.

Benzodiazepine-derived anxiolytics (N05BA) show high percentages in Torrecilla (6.22%) and San Román (7.39%). San Román also stands out for the dispensation of medications for cardiovascular pathologies, such as platelet aggregation inhibitors (B01AC), selective beta blockers (C07AB), and statins (C10AA). In Murillo, there is a notable percentage in the dispensation of antibiotics from the families J01CR (4.33%) and J01FA (3.80%)

**FIGURE 3:**
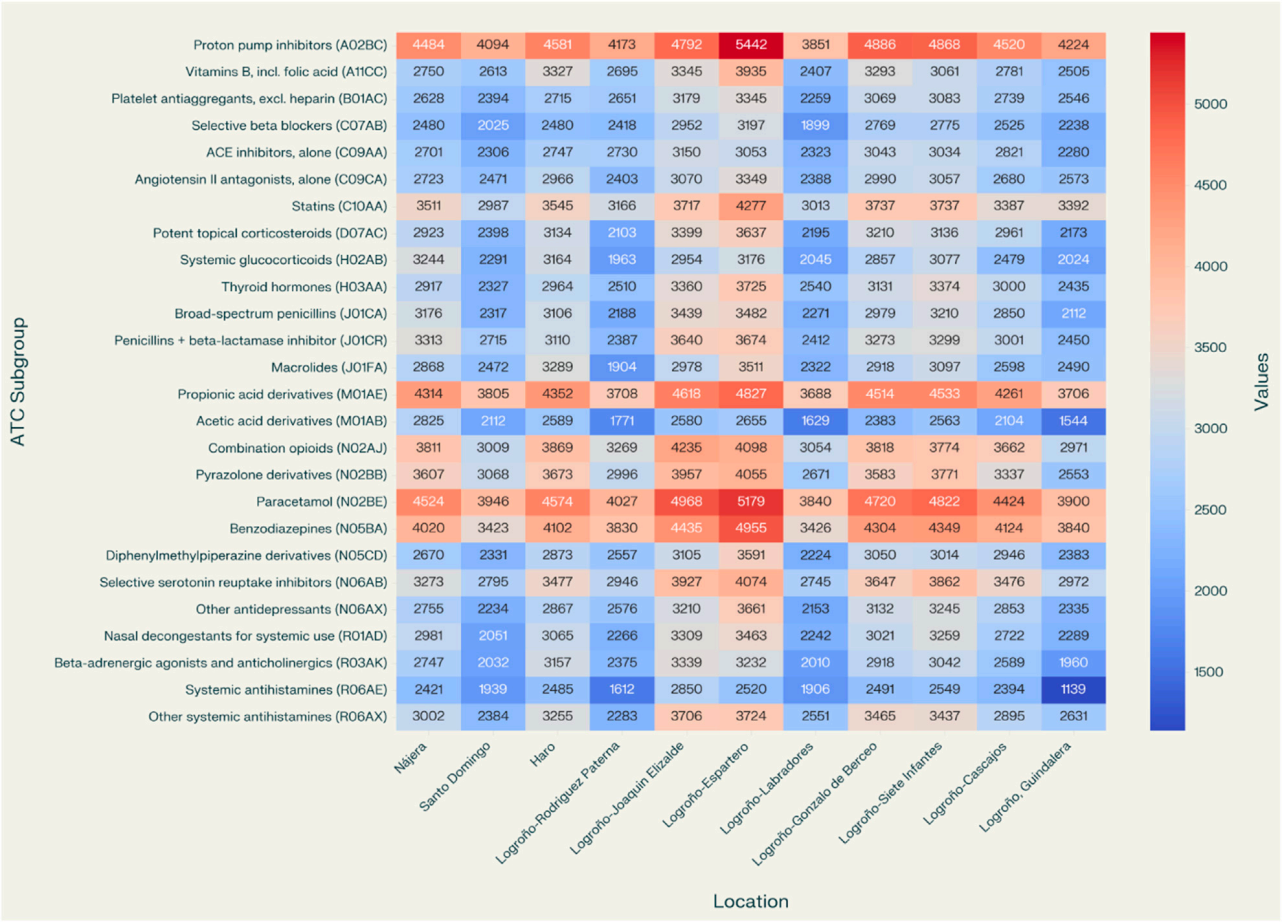
Distribution of medication dispensation in the basic health zones of “cluster Rioja-2”.

The analysis of the distribution of medication dispensation in the cluster named RIOJA-2, composed of the basic health zones of Nájera, Santo Domingo, Haro, Logroño-Rodríguez Paterna, Logroño-Joaquín Elizalde, Logroño-Espartero, Logroño-Labradores, Logroño-Gonzalo de Berceo, Logroño-Siete Infantes, Logroño-Cascajos, and Logroño-Guindalera, generally shows great homogeneity in terms of the percentages of the different ATC subgroups. However, it is worth noting that the subgroup C10AA (HMG CoA reductase inhibitors) has a high percentage in the Logroño-Guindalera area (5.01%). The family of analgesics derived from propionic acid (M01AE) has a significant percentage in the Nájera area (3.42%), with 2,825 dispensations. Finally, the subgroup N05BA (benzodiazepine-derived anxiolytics) stands out with significant percentages in the Rodriguez Paterna area of Logroño (5.51%) and the Guindalera area of Logroño (5.68%). See Tables 4, 5 and Figure 3.

**TABLE 4:** Distribution of medication dispensation in the basic health zones of “cluster Rioja-1”.

ATC4 (name) number of individual health card (IHC)	Cervera	Alfaro	Calahorra	Arnedo	Murillo	San román	Alberite	Torrecilla	Navarrete	Total
A02BC Proton pump inhibitors	2,855	4,418	5,509	4,647	3,401	692	4,643	1,583	3,983	82,195
%	7.19%	5.65%	5.47%	5.62%	6.32%	9.56%	5.36%	8.17%	5.58%	5.73%
A11CC Vitamin D and analogues	1,287	3,263	3,578	3,204	2,156	99	3,032	432	2,591	52,585
%	3.24%	4.17%	3.55%	3.88%	4.01%	1.37%	3.50%	2.23%	3.63%	3.67%
B01AC Platelet aggregation inhibitors, excl. Heparin	1,578	2,523	3,441	2,771	1,775	350	2,936	794	2,324	49,229
%	3.97%	3.22%	3.42%	3.35%	3.30%	4.84%	3.39%	4.10%	3.26%	3.43%
C07AB Beta blocking agents, selective	1,340	2,608	3,200	2,643	1,608	247	2,547	597	2,205	44,835
%	3.37%	3.33%	3.18%	3.20%	2.99%	3.41%	2.94%	3.08%	3.09%	3.13%
C09AA ACE inhibitors, plain	1,491	2,322	3,267	2,609	1,840	278	2,834	861	2,638	48,403
%	3.75%	2.97%	3.24%	3.16%	3.42%	3.84%	3.27%	4.45%	3.70%	3.38%
C09CA Angiotensin II receptor blockers	1,346	2,690	3,443	2,696	1,703	103	2,952	632	2,350	48,677
%	3.39%	3.44%	3.42%	3.26%	3.16%	1.42%	3.41%	3.26%	3.29%	3.40%
C10AA HMG CoA reductase inhibitors	2,260	3,182	4,345	3,614	2,480	863	3,602	1,343	3,171	63,538
%	5.69%	4.07%	4.32%	4.37%	4.61%	11.93%	4.16%	6.94%	4.45%	4.43%
D07AC Corticosteroids, potent (group III)	1,116	2,618	3,586	2,676	1,726	123	3,141	494	2,447	49,324
%	2.81%	3.35%	3.56%	3.24%	3.21%	1.70%	3.63%	2.55%	3.43%	3.44%
H02AB Glucocorticoids	1,199	2,748	3,452	2,740	1,968	100	3,074	423	2,711	47,942
%	3.02%	3.51%	3.43%	3.32%	3.66%	1.38%	3.55%	2.18%	3.80%	3.34%
H03AA Thyroid hormones	1,173	2,797	3,567	2,821	1,571	119	2,896	618	2,448	50,412
%	2.95%	3.57%	3.54%	3.41%	2.92%	1.64%	3.35%	3.19%	3.43%	3.52%
J01CA Penicillin with extended spectrum	1,144	2,870	3,540	2,876	1,332	170	3,125	481	2,420	49,936
%	2.88%	3.67%	3.52%	3.48%	2.47%	2.35%	3.61%	2.48%	3.39%	3.48%
J01CR Combinations of penicillins, incl. Beta lactamase inhibitors	1,357	2,941	3,927	3,265	2,331	254	3,497	615	2,592	54,854
%	3.42%	3.76%	3.90%	3.95%	4.33%	3.51%	4.04%	3.18%	3.63%	3.83%
J01FA Macrolides	1,677	2,811	3,617	2,856	2,045	48	2,919	462	2,239	49,449
%	4.22%	3.59%	3.59%	3.46%	3.80%	0.66%	3.37%	2.39%	3.14%	3.45%
M01AB acetic acid derivatives and related substances	1,105	2,627	3,310	2,341	1,484	79	2,357	496	2,118	40,951
%	2.78%	3.36%	3.29%	2.83%	2.76%	1.09%	2.72%	2.56%	2.97%	2.86%
M01AE Propionic acid derivatives	2,510	4,177	5,056	4,359	3,249	415	4,592	1,388	3,867	77,006
%	6.32%	5.34%	5.02%	5.27%	6.04%	5.74%	5.30%	7.17%	5.42%	5.37%
N02AJ Opioids in combination with non-opioid analgesics	1,903	3,735	4,446	3,736	2,441	328	3,890	1,073	3,444	64,855
%	4.79%	4.77%	4.42%	4.52%	4.54%	4.53%	4.49%	5.54%	4.83%	4.52%
N02BB Pyrazolones	1,626	3,370	4,335	3,494	2,565	423	3,951	969	3,206	61,438
%	4.09%	4.31%	4.31%	4.23%	4.77%	5.85%	4.56%	5.00%	4.49%	4.29%
N02BE Anilides	2,581	4,194	5,211	4,432	3,457	774	4,760	1,366	3,914	80,131
%	6.50%	5.36%	5.18%	5.36%	6.42%	10.70%	5.50%	7.05%	5.49%	5.59%
N05BA Benzodiazepine derivatives. Anxiolytics	2,059	3,818	4,655	3,958	2,736	535	4,169	1,205	3,589	71,932
%	5.18%	4.88%	4.62%	4.79%	5.08%	7.39%	4.82%	6.22%	5.03%	5.02%
N05CD Benzodiazepine derivatives. Hypnotics and sedatives	1,140	2,389	3,312	2,712	1,544	151	3,038	520	2,448	48,156
%	2.87%	3.05%	3.29%	3.28%	2.87%	2.09%	3.51%	2.69%	3.43%	3.36%
N06AB Selective serotonin reuptake inhibitors	1,556	2,936	3,837	3,312	2,195	362	3,495	775	2,876	58,690
%	3.92%	3.75%	3.81%	4.01%	4.08%	5.00%	4.04%	4.00%	4.03%	4.09%
N06AX Other antidepressants	1,259	2,553	3,548	2,949	1,868	209	3,025	545	2,333	49,443
%	3.17%	3.26%	3.52%	3.57%	3.47%	2.89%	3.49%	2.81%	3.27%	3.45%
R01AD Corticosteroids	880	2,800	3,614	3,155	1,555	100	3,128	465	2,361	48,889
%	2.22%	3.58%	3.59%	3.82%	2.89%	1.38%	3.61%	2.40%	3.31%	3.41%
R03AK Adrenergics in combination with corticosteroids or other drugs, excl. Anticholinergics	1,207	2,839	3,844	3,027	1,493	130	3,064	472	2,101	47,650
%	3.04%	3.63%	3.82%	3.66%	2.77%	1.80%	3.54%	2.44%	2.95%	3.32%
R06AE Piperazine derivatives	678	2,185	3,154	2,782	1,356	148	2,629	256	2,133	39,739
%	1.71%	2.79%	3.13%	3.37%	2.52%	2.05%	3.04%	1.32%	2.99%	2.77%
R06AX Other antihistamines for systemic use	1,388	2,843	3,891	2,968	1,943	135	3,266	499	2,824	53,272
%	3.49%	3.63%	3.86%	3.59%	3.61%	1.87%	3.77%	2.58%	3.96%	3.72%
Total	39,715	78,257	100,685	82,643	53,822	7,235	86,562	19,364	71,333	1,433,531
%	100.00%	100.00%	100.00%	100.00%	100.00%	100.00%	100.00%	100.00%	100.00%	100.00%

**TABLE 5:** Distribution of medication dispensation in the basic health zones of “cluster Rioja-2.

ATC 4	Nájera	Santo domingo	Haro	Logroño-rodriguez paterna	Logroño-joaquín elizalde	Logroño-espartero	Logroño-labradores	Logroño-gonzalo de berceo	Logroño-siete infantes	Logroño-cascajos	Logroño, guindalera	Total
A02BC Proton pump inhibitors	4,484	4,094	4,581	4,173	4,792	5,442	3,851	4,886	4,868	4,520	4,224	82,195
%	5.42	5.97	5.36	6.00	5.20	5.56	5.83	5.60	5.47	5.64	6.24	5.73
A11CC Vitamin D and analogues	2,750	2,613	3,327	2,695	3,345	3,935	2,407	3,293	3,061	2,781	2,505	52,585
%	3.33	3.81	3.89	3.88	3.63	4.02	3.64	3.78	3.44	3.47	3.70	3.67
B01AC Platelet aggregation inhibitors, excl. Heparin	2,628	2,394	2,715	2,651	3,179	3,345	2,259	3,069	3,083	2,739	2,546	49,229
%	3.18	3.49	3.18	3.81	3.45	3.42	3.42	3.52	3.46	3.42	3.76	3.43
C07AB Beta blocking agents, selective	2,480	2,025	2,480	2,418	2,952	3,197	1,899	2,769	2,775	2,525	2,238	44,835
%	3.00	2.95	2.90	3.48	3.20	3.27	2.87	3.18	3.12	3.15	3.31	3.13
C09AA ACE inhibitors, plain	2,701	2,306	2,747	2,730	3,150	3,053	2,323	3,043	3,034	2,821	2,280	48,403
%	3.27	3.36	3.21	3.93	3.42	3.12	3.52	3.49	3.41	3.52	3.37	3.38
C09CA Angiotensin II receptor blockers	2,723	2,471	2,966	2,403	3,070	3,349	2,388	2,990	3,057	2,680	2,573	48,677
%	3.29	3.61	3.47	3.46	3.33	3.42	3.61	3.43	3.43	3.34	3.80	3.40
C10AA HMG CoA reductase	3,511	2,987	3,545	3,166	3,717	4,277	3,013	3,737	3,737	3,387	3,392	63,538
%	4.25	4.36	4.15	4.55	4.03	4.37	4.56	4.29	4.20	4.23	5.01	4.43
D07AC Corticosteroids, potent (group III)	2,923	2,398	3,134	2,103	3,399	3,637	2,195	3,210	3,136	2,961	2,173	49,324
%	3.54	3.50	3.67	3.03	3.69	3.72	3.32	3.68	3.52	3.70	3.21	3.44
H02AB Glucocorticoids	3,244	2,291	3,164	1,963	2,954	3,176	2,045	2,857	3,077	2,479	2,024	47,942
%	3.92	3.34	3.70	2.82	3.20	3.25	3.10	3.28	3.46	3.09	2.99	3.34
H03AA Thyroid hormones	2,917	2,327	2,964	2,510	3,360	3,725	2,540	3,131	3,374	3,000	2,435	50,412
%	3.53	3.40	3.47	3.61	3.64	3.81	3.84	3.59	3.79	3.74	3.60	3.52
J01CA Penicillin with extended spectrum	3,176	2,317	3,106	2,188	3,439	3,482	2,271	2,979	3,210	2,850	2,112	49,936
%	3.84	3.38	3.63	3.15	3.73	3.56	3.44	3.42	3.61	3.56	3.12	3.48
J01CR Combinations of penicillins, incl. Beta lactamase inhibitors	3,313	2,715	3,110	2,387	3,640	3,674	2,412	3,273	3,299	3,001	2,450	54,854
%	4.01	3.96	3.64	3.43	3.95	3.76	3.65	3.75	3.71	3.75	3.62	3.83
J01FA Macrolides	2,868	2,472	3,289	1,904	2,978	3,511	2,322	2,918	3,097	2,598	2,490	49,449
%	3.47	3.61	3.85	2.74	3.23	3.59	3.51	3.35	3.48	3.24	3.68	3.45
M01AB acetic acid derivatives and related substances	4,314	3,805	4,352	3,708	4,618	4,827	3,688	4,514	4,533	4,261	3,706	77,006
%	5.22	5.55	5.09	5.33	5.01	4.93	5.58	5.18	5.09	5.32	5.48	5.37
M01AE Propionic acid derivatives	2,825	2,112	2,589	1,771	2,580	2,655	1,629	2,383	2,563	2,104	1,544	40,951
%	3.42	3.08	3.03	2.55	2.80	2.71	2.47	2.73	2.88	2.63	2.28	2.86
N02AJ Opioids in combination with non-opioid analgesics	3,811	3,009	3,869	3,269	4,235	4,098	3,054	3,818	3,774	3,662	2,971	64,855
%	4.61	4.39	4.53	4.70	4.59	4.19	4.62	4.38	4.24	4.57	4.39	4.52
N02BB Pyrazolones	3,607	3,068	3,673	2,996	3,957	4,055	2,671	3,583	3,771	3,337	2,553	61,438
%	4.36	4.48	4.30	4.31	4.29	4.14	4.04	4.11	4.24	4.16	3.77	4.29
N02BE Anilides	4,524	3,946	4,574	4,027	4,968	5,179	3,840	4,720	4,822	4,424	3,900	80,131
%	5.47	5.76	5.35	5.79	5.39	5.29	5.81	5.41	5.42	5.52	5.76	5.59
N05BA Benzodiazepine derivatives. Anxiolytics	4,020	3,423	4,102	3,830	4,435	4,955	3,426	4,304	4,349	4,124	3,840	71,932
%	4.86	4.99	4.80	5.51	4.81	5.06	5.19	4.94	4.88	5.15	5.68	5.02
N05CD Benzodiazepine derivatives. Hypnotics and sedatives	2,670	2,331	2,873	2,557	3,105	3,591	2,224	3,050	3,014	2,946	2,383	48,156
%	3.23	3.40	3.36	3.68	3.37	3.67	3.37	3.50	3.39	3.68	3.52	3.36
N06AB Selective serotonin reuptake inhibitors	3,273	2,795	3,477	2,946	3,927	4,074	2,745	3,647	3,862	3,476	2,972	58,690
%	3.96	4.08	4.07	4.24	4.26	4.16	4.16	4.18	4.34	4.34	4.39	4.09
N06AX Other antidepressants	2,755	2,234	2,867	2,576	3,210	3,661	2,153	3,132	3,245	2,853	2,335	49,443
%	3.33	3.26	3.35	3.71	3.48	3.74	3.26	3.59	3.64	3.56	3.45	3.45
R01AD Corticosteroids	2,981	2,051	3,065	2,266	3,309	3,463	2,242	3,021	3,259	2,722	2,289	48,889
%	3.61	2.99	3.59	3.26	3.59	3.54	3.39	3.46	3.66	3.40	3.38	3.41
R03AK Adrenergics in combination with corticosteroids or other drugs, excl. Anticholinergics	2,747	2,032	3,157	2,375	3,339	3,232	2,010	2,918	3,042	2,589	1,960	47,650
%	3.32	2.96	3.69	3.42	3.62	3.30	3.04	3.35	3.42	3.23	2.90	3.32
R06AE Piperazine derivatives	2,421	1,939	2,485	1,612	2,850	2,520	1,906	2,491	2,549	2,394	1,139	39,739
%	2.93	2.83	2.91	2.32	3.09	2.58	2.89	2.86	2.86	2.99	1.68	2.77
R06AX Other antihistamines for systemic use	3,002	2,384	3,255	2,283	3,706	3,724	2,551	3,465	3,437	2,895	2,631	53,272
%	3.63	3.48	3.81	3.28	4.02	3.81	3.86	3.97	3.86	3.61	3.89	3.72
Total	82,668	68,539	85,466	69,507	92,214	97,837	66,064	87,201	89,028	80,129	67,665	1,433,531
%	100.00	100.00	100.00	100.00	100.00	100.00	100.00	100.00	100.00	100.00	100.00	100.00

Regarding medication dispensations according to IHC (Individual Health Card) status, various significant trends are identified. In the analysis of dispensations for users who pay 100% of medication costs (NOFAR), propionic acid derivatives (M01AE) lead the percentage of dispensations (16.67%), followed by Proton Pump Inhibitors (A02BC) and Paracetamol and its combinations (N02BE), both at 8.14%. For IHC 001 (vulnerable groups, free pharmacy), the most dispensed medications are Paracetamol and its combinations (N02BE) with 16,374 dispensations (6.40%), followed by Proton Pump Inhibitors (A02BC) with 16,107 dispensations (6.29%) and Propionic Acid Derivatives (M01AE) with 14,628 dispensations (5.71%).

For IHC 002 (pensioners), Proton Pump Inhibitors (A02BC) stand out with 31,419 dispensations (5.71%), Paracetamol (N02BE) with 30,093 dispensations (5.47%), and Benzodiazepines (N05BA) with 27,935 dispensations (5.08%). In IHC 003 (low-income workers), the most consumed medications are Propionic Acid Derivatives (M01AE) with 16,950 dispensations (5.05%), followed by Proton Pump Inhibitors (A02BC) with 16,849 dispensations (5.02%) and Paracetamol (N02BE) with 16,842 dispensations (5.02%).

For IHC 004 (medium-income workers), higher dispensation of Propionic Acid Derivatives (M01AE) is observed with 14,458 dispensations (6.01%), Paracetamol (N02BE) with 12,890 dispensations (5.36%), and Proton Pump Inhibitors (A02BC) with 12,454 dispensations (5.18%). For IHC 005 (high-income workers), Statins (C10AA) lead dispensation with 3,579 dispensations (12.58%), followed by Proton Pump Inhibitors (A02BC) with 3,294 dispensations (11.58%) and Benzodiazepines (N05BA) with 2,122 dispensations (7.46%).

Finally, for IHC 006 (mutualist civil servants), Statins (C10AA) stand out with 22 dispensations (11.64%), followed by Benzodiazepines (N05BA) with 15 dispensations (7.94%) and Propionic Acid Derivatives (M01AE) with 13 dispensations (6.88%). This analysis highlights how therapeutic needs and medication dispensation vary according to the economic and social situation of users, revealing specific patterns in each IHC group. See Table 6.

**TABLE 6:** Distribution of medication dispensation by income and IHC status.

ATC4	NOFAR	%	IHC 001	%	IHC 002	%	IHC 003	%	IHC 004	%	IHC 005	%	IHC 006	%	Total	%
A02BC Proton pump inhibitors	21	8.14	16,107	6.29	31,419	5.71	16,849	5.02	12,454	5.18	3,294	11.58	9	4.76	82,195	5.73
A11CC Vitamin D and analogues	3	1.16	9,851	3.85	21,732	3.95	11,384	3.39	7,694	3.20	1,050	3.69	2	1.06	52,585	3.67
B01AC Platelet aggregation inhibitors, excl. Heparin	11	4.26	8,782	3.43	23,511	4.27	9,173	2.73	5,994	2.49	1,230	4.32	4	2.12	49,229	3.43
C07AB Beta blocking agents, selective	7	2.71	7,719	3.02	21,060	3.83	8,557	2.55	5,986	2.49	1,159	4.07	11	5.82	44,835	3.13
C09AA ACE inhibitors, plain	4	1.55	8,485	3.31	21,828	3.97	9,918	2.96	6,755	2.81	970	3.41	3	1.59	48,403	3.38
C09CA Angiotensin II receptor blockers	11	4.26	8,193	3.20	21,967	3.99	9,287	2.77	6,904	2.87	1,611	5.66	13	6.88	48,677	3.40
C10AA HMG CoA reductase	6	2.33	11,576	4.52	26,800	4.87	11,703	3.49	8,926	3.71	3,579	12.58	22	11.64	63,538	4.43
D07AC Corticosteroids, potent (group III)	7	2.71	8,709	3.40	17,500	3.18	13,218	3.94	8,749	3.64	346	1.22	4	2.12	49,324	3.44
H02AB Glucocorticoids	4	1.55	7,598	2.97	18,734	3.41	12,283	3.66	8,293	3.45	585	2.06	4	2.12	47,942	3.34
H03AA Thyroid hormones	8	3.10	8,453	3.30	19,087	3.47	12,014	3.58	9,018	3.75	994	3.49	12	6.35	50,412	3.52
J01CA Penicillin with extended spectrum	9	3.49	8,536	3.33	16,227	2.95	14,070	4.19	9,880	4.11	417	1.47	5	2.65	49,936	3.48
J01CR Combinations of penicillins, incl. Beta lactamase inhibitors	10	3.88	9,716	3.80	18,250	3.32	14,898	4.44	10,598	4.41	474	1.67	3	1.59	54,854	3.83
J01FA Macrolides	4	1.55	7,351	2.87	16,703	3.04	13,906	4.15	10,285	4.28	630	2.21	5	2.65	49,449	3.45
M01AB acetic acid derivatives and related substances	6	2.33	6,377	2.49	13,167	2.39	12,550	3.74	7,835	3.26	308	1.08	1	0.53	40,951	2.86
M01AE Propionic acid derivatives	43	16.67	14,628	5.71	26,422	4.80	16,950	5.05	14,458	6.01	1,541	5.42	13	6.88	77,006	5.37
N02AJ Opioids in combination with non-opioid analgesics	12	4.65	12,017	4.69	23,937	4.35	15,452	4.61	11,447	4.76	858	3.02	5	2.65	64,855	4.52
N02BB Pyrazolones	13	5.04	12,134	4.74	23,531	4.28	14,518	4.33	9,467	3.94	632	2.22	5	2.65	61,438	4.29
N02BE Anilides	21	8.14	16,374	6.40	30,093	5.47	16,842	5.02	12,890	5.36	1,587	5.58	10	5.29	80,131	5.59
N05BA Benzodiazepine derivatives. Anxiolytics	13	5.04	14,220	5.56	27,935	5.08	15,280	4.56	11,361	4.72	2,122	7.46	15	7.94	71,932	5.02
N05CD Benzodiazepine derivatives. Hypnotics and sedatives	4	1.55	9,113	3.56	22,293	4.05	9,692	2.89	5,960	2.48	853	3.00	4	2.12	48,156	3.36
N06AB Selective serotonin reuptake inhibitors	5	1.94	10,501	4.10	23,155	4.21	13,459	4.01	9,904	4.12	1,237	4.35	12	6.35	58,690	4.09
N06AX Other antidepressants	10	3.88	9,107	3.56	21,803	3.96	10,533	3.14	6,987	2.91	742	2.61	4	2.12	49,443	3.45
R01AD Corticosteroids	7	2.71	7,405	2.89	15,755	2.86	13,775	4.11	10,626	4.42	664	2.33	8	4.23	48,889	3.41
R03AK Adrenergics in combination with corticosteroids or other drugs, excl. Anticholinergics	3	1.16	8,382	3.27	18,160	3.30	12,170	3.63	7,973	3.32	632	2.22	5	2.65	47,650	3.32
R06AE Piperazine derivatives	7	2.71	6,007	2.35	12,093	2.20	12,404	3.70	8,323	3.46	252	0.89	1	0.53	39,739	2.77
R06AX Other antihistamines for systemic use	9	3.49	8,638	3.37	16,834	3.06	14,545	4.34	11,679	4.86	677	2.38	9	4.76	53,272	3.72
Total	258	100.00	255,979	100.00	549,996	100.00	335,430	100.00	240,446	100.00	28,444	100.00	189	100.00	1,433,531	100.00

The IHC system classifies users into categories based on their income and economic situation to regulate medication copayments, promoting equity and social justice: IHC 001: Exempt from contribution: unemployed individuals who have lost the right to unemployment benefits, recipients of social integration income, individuals affected by toxic syndrome and people with disabilities, recipients of non-contributory pensions, beneficiaries of the minimum living income, recipients of Social Security economic benefits for children or minors in foster care, pensioners with an annual income below €5,635. IHC 002: Contribute 10% with a monthly limit based on their income: pensioners and their beneficiaries with incomes greater than or equal to €5,635 and less than €100,000. IHC 003: Contribution of 40%: insured individuals with incomes below €18,000 and their beneficiaries. IHC 004: Contribution of 50%: insured individuals with incomes below €100,000 and greater than or equal to €18,000 and their beneficiaries. Workers with intermediate incomes, copayment of 50%. IHC 005: Contribution of 60%: active insured individuals or pensioners with incomes equal to or greater than €100,000 and their beneficiaries. IHC 006: Contribution of 30%: insured individuals covered by mutual societies. NOFAR: Pay 100% of the medication price: individuals with special agreements.

## 4 Discussion

The objective of this research is to evaluate the status of medication dispensation in La Rioja and establish its relationship with health determinants such as economic conditions, area of residence, age, and gender. The most prescribed medication subgroups in La Rioja are led by proton pump inhibitors, followed by two families of analgesics (propionic acid derivatives and anilides) and benzodiazepine-derived anxiolytics. These results align with those presented in the Pharmaceutical Benefits Report of 2023 prepared by the Ministry of Health (Author anonymous, 2023), although in this case, they refer to dispensation in terms of the number of packages. Regarding the notable increase observed in La Rioja in the dispensation of the vitamin D and analogs subgroup (A11CC), this fact coincides with the results presented in various studies conducted in Spain, which reflect significant increases in these treatments and warn of a high number of patients for whom such treatments are not justified (Abril Rubio et al., 2022).

Another significant increase is observed in the antidepressants subgroup N06AX. This result coincides with the study conducted in Barakaldo-Sestao, which shows a significant increase in antidepressant dispensations during the pandemic, with an even more pronounced increase afterward. In the same study, it was observed that the consequences of the pandemic translated into a considerable impact on emotional health, which manifested in the following years (Martinez-Cengotitabengoa et al., 2025).

Regarding the most prescribed medication subgroups in older age groups, medications indicated for the treatment of cardiovascular diseases predominate, such as platelet aggregation inhibitors, selective beta-blockers, and angiotensin II receptor blockers. These treatments are usually chronic and habitual in older individuals (Legrain and Lacaille, 2005), unlike those identified in younger age groups, such as certain antibiotics (J01CA), antihistamines, and analgesics, which are usually indicated for acute treatments.

Differences have been observed in medication dispensation according to gender. In general, dispensation rates were higher in women, which aligns with studies indicating a higher prevalence of medication dispensation in this gender. This may be explained by greater medical attendance and, therefore, a higher probability of detection and diagnosis of pathologies (Sans et al., 2002) (Skoog et al., 2014). In our study, differences have been detected in the dispensation of certain medication families according to gender. The subgroups most differentiated in favor of women include thyroid hormones (H03AA), vitamin D and its analogs, which align with studies showing a higher prevalence of conditions such as hypothyroidism and osteoporosis in women (Escribano-Serrano et al., 2014) (Hermoso de Mendoza, 2003). Furthermore, there is also a significant difference in favor of women in the number of antidepressant dispensations. This fact aligns with findings in some studies that argue for an unequal position of women, stemming from more precarious living conditions that generate greater mental suffering (Bacigalupe et al., 2022).

Regarding anxiolytics, our study shows a higher dispensing rate for benzodiazepine-derived anxiolytics (N05BA) and benzodiazepine-derived hypnotic-sedatives in women. These results align with those presented in the Primary Care Clinical Database (BDCAP) (Ministerio de Sanidad, 2018), which shows a higher prevalence of anxiety disorders in women. There are also studies related to health determinants indicating that depressive disorders are one of the health problems that most affect women’s quality of life, occurring twice as frequently in women as in men in our country (Author anonymous, 2024). The same study also highlights pathologies that predominantly affect women, such as lower back pain, which is the leading cause of temporary work disability in Spain. In this regard, another study associated back pain primarily with the female gender and cites diclofenac and paracetamol among the most commonly used medications for this condition (Bassols et al., 2025).

Our study shows that the number of dispensations for analgesics from the acetic acid derivatives family (M01AB), such as diclofenac, was very similar in all genders, although the percentage was slightly higher in men. The families of other analgesic groups, such as propionic acid derivatives, opioids, pyrazolones, or anilides, showed a higher number of dispensations in women, and in terms of percentages, they were very similar between sexes.

Regarding diseases that most affect men’s quality of life, such as cardiovascular disease (Author anonymous, 2024), which also has a higher prevalence—in global terms—in men (Ministerio de Sanidad, 2025), our study shows a series of medication groups indicated for this pathology, such as platelet aggregation inhibitors (B01AC), selective beta-blockers (C07AB), and angiotensin-converting enzyme inhibitors (C09AA), where the number of dispensations and percentages are higher in men.

Regarding income level measured by IHC (Individual Health Card), it is observed that most dispensations (38.4%) belong to pensioner users with IHC 2. This fact may be due to this group consisting of older individuals, thus consuming more medication (Valderrama et al., 1998). Medications from the proton pump inhibitor family (A02BC) appear in a high proportion across all IHC groups, although they have a particularly high percentage in IHC 5 (11.58%). Regarding the dispensing of anxiolytics (families N05BA and N05CD), high percentages are observed in groups with low-income levels, IHC 1, and pensioners with IHC 2. These data align with a study by the Ministry showing higher dispensation of these drugs in unemployed individuals and those with low-income levels (Ministerio de Sanidad, 2023b).

Regarding antibiotic dispensation, it is observed that the population with higher income levels, IHC 5, was dispensed fewer antibiotics than the rest of the IHC groups, as is the case with the J01CA group of penicillins, with a percentage of 1.47%. This result coincides with Ministry data (Caracterización del consumo de antibióticos, 2025). The study of factors influencing antibiotic use in primary care indicates that population income influences antibiotic use, as patients with higher incomes may frequent public centers less in favor of visiting private centers with private dispensations (Atención Primaria et al., 1999).

In the case of IHC 003 (workers with low incomes), the most consumed medications are propionic acid derivatives (M01AE) with 16,950 dispensations (5.05%), followed by PPIs (A02BC) with 16,849 dispensations (5.02%) and paracetamol (N02BE) with 16,842 dispensations (5.02%). These results align with the evidence, as individuals with symptomatic and disabling disorders are more motivated to initiate treatment, according to Gil-Girbau et al. (Gil-Girbau et al., 2020). Additionally, these conditions may lead to significant productivity losses due to sick leave and unemployment, increasing the vulnerability of those suffering from pain, as noted by Giladi et al. (Giladi et al., 2015). Furthermore, workers with low wages and worse working conditions are at greater risk of developing pain-related disorders and face barriers to accessing quality care, which may hinder their recovery, according to Frederiksen et al. (Frederiksen et al., 2015).

Regarding population residence, greater homogeneity is observed in the different medication families in cluster 2. This may be due to the rural population being numerically more similar to the urban population. However, in cluster 1, there is a greater difference in the number of inhabitants in the municipalities. Small and rural towns, such as San Román and Torrecilla, have a high percentage of benzodiazepine-derived anxiolytics (N05BA), ranging from 6.22% to 7.39%. These data align with the Ministry study, which notes higher use of these medications in small municipalities (Ministerio de Sanidad, 2023b). The high dispensing rate of statins in San Román may be explained by population aging. Regarding antibiotic dispensation, the Murillo area stands out in dispensations of macrolides (J01FA) at 3.80% and penicillin combinations (J01CR) at 4.33%, possibly due to higher medical consultation rates or the aging of this population (Serna et al., 2011).

## 5 Conclusion

Proton pump inhibitors, anti-inflammatory drugs derived from propionic acid, analgesics containing paracetamol, and benzodiazepine-derived anxiolytics are the most prescribed medications in La Rioja, following national and European trends.

The dispensation of vitamin D and antidepressants has increased significantly, especially among women, associated with population aging and gender differences.

Socioeconomic determinants, such as low income and unemployment, directly influence access to and dispensation of medications, highlighting vulnerabilities among pensioners and workers with limited incomes. Areas with higher population aging show increased dispensation of medications for chronic diseases, reflecting regional health inequalities.

## 6 Limitations and strengths

This study has several limitations that should be considered when interpreting the findings. First, its observational design does not allow causal inference; it provides a descriptive mapping of dispensation patterns within a defined 2016–2023 timeframe. Second, dispensations linked to civil servant mutual insurance schemes excluded from the National Health System under IHC 006 are likely underrepresented, as many beneficiaries obtain care through private providers. Third, the analysis was restricted to the 26 most frequent ATC level 4 subgroups, selected through an objective cumulative-volume and temporal-consistency criterion. While this approach enhances statistical stability and policy relevance, it may overlook emerging trends in less prevalent subgroups or in specific active substances (ATC level 5).

This is a descriptive study focused on user prevalence (patient–ATC4–year; at least one dispensing), not consumption intensity. DID was not used to maintain design coherence, which limits direct comparisons with dose-standardized indicators (e.g., Spanish Ministry of Health, OECD). In planned future work (Phase 2), we will incorporate DID with appropriate methodological harmonization.

Despite these constraints, the study has notable strengths. It provides a comprehensive and faithfully curated overview of medication dispensation in La Rioja over eight consecutive years, integrating key social determinants (age, sex, territorial context, and socioeconomic factors). This equity-oriented framing enhances its utility for strategic health system planning—informing resource allocation, targeting inequities, and supporting rational use interventions. Moreover, by defining a clear analytical unit (patient–ATC4 subgroup–year) and minimizing duplication, the study establishes a reproducible platform for longitudinal monitoring. The resulting dataset and methodological framework create a robust foundation for future research exploring dose-adjusted utilization, temporal shifts, and interaction effects between demographic and socioeconomic determinants, thereby amplifying its potential contribution to public health decision-making.

## Data Availability

The raw data will be available upon request from the first author.
